# Development of a Multi-Enzymatic Biocatalytic System through Immobilization on High Quality Few-Layer bio-Graphene

**DOI:** 10.3390/nano13010127

**Published:** 2022-12-26

**Authors:** Christina Alatzoglou, Michaela Patila, Archontoula Giannakopoulou, Konstantinos Spyrou, Feng Yan, Wenjian Li, Nikolaos Chalmpes, Angeliki C. Polydera, Petra Rudolf, Dimitrios Gournis, Haralambos Stamatis

**Affiliations:** 1 Laboratory of Biotechnology, Department of Biological Applications and Technology, University of Ioannina, 45110 Ioannina, Greece; 2 Department of Materials Science & Engineering, University of Ioannina, 45110 Ioannina, Greece; 3 Zernike Institute for Advanced Materials, University of Groningen, Nijenborgh 4, 9747 AG Groningen, The Netherlands; 4 Engineering and Technology Institute Groningen, University of Groningen, Nijenborgh 4, 9747 AG Groningen, The Netherlands

**Keywords:** bio-Graphene, bovine serum albumin, green synthesis, nanobiocatalytic system, multi-enzymatic immobilization, cascade reactions

## Abstract

In this work, we report the green production of few-layer bio-Graphene (bG) through liquid exfoliation of graphite in the presence of bovine serum albumin. Microscopic characterization evaluated the quality of the produced nanomaterial, showing the presence of 3–4-layer graphene. Moreover, spectroscopic techniques also confirmed the quality of the resulted bG, as well as the presence of bovine serum albumin on the graphene sheets. Next, for the first time, bG was used as support for the simultaneous covalent co-immobilization of three enzymes, namely *β*-glucosidase, glucose oxidase, and horseradish peroxidase. The three enzymes were efficiently co-immobilized on bG, demonstrating high immobilization yields and activity recoveries (up to 98.5 and 90%, respectively). Co-immobilization on bG led to an increase of apparent *K*_M_ values and a decrease of apparent *V*_max_ values, while the stability of the nanobiocatalysts prevailed compared to the free forms of the enzymes. Co-immobilized enzymes exhibited high reusability, preserving a significant part of their activity (up to 72%) after four successive catalytic cycles at 30 °C. Finally, the tri-enzymatic nanobiocatalytic system was applied in three-step cascade reactions, involving, as the first step, the hydrolysis of *p*-Nitrophenyl-*β*-D-Glucopyranoside and cellobiose.

## 1. Introduction

Graphene, a single sheet of carbon atoms that are bonded in a hexagonal arrangement, has been extensively researched since its discovery, due to its amazing characteristics, such as high electrical and thermal conductivity, and mechanical strength [1]. These features have put this nanomaterial in the scientific spotlight, offering applications in a variety of technological and scientific fields, from the development of bio-sensors [2], to the synthesis of nanocomposite materials [3], and the production of solar [4] and fuel cells [5]. The synthesis of pristine graphene can be achieved via micromechanical cleavage [6], chemical vapor deposition (CVD) [7], liquid-phase exfoliation [8], and epitaxial growth [9]. Compared to these approaches, liquid-phase ultrasonic exfoliation is a low cost and highly versatile way to produce defect-poor graphene [8]. A plethora of chemical compounds can be utilized to exfoliate graphite and stabilize the layers of graphene, based on their ionic size and how easily they intercalate. However, the majority of molecules traditionally employed for intercalation, such as acids, bases, and ionic liquids, are often hazardous and not environmentally safe; moreover, they require a relatively high energy input [10]. The biocompatibility of the end product, along with its quality, cost, and environmental impact of the procedures, has yet to be addressed. As a consequence, the search of an environmentally friendly route for the production of graphene is emerging as highly important.

Towards this aim, the use of biomolecules, such as proteins, for liquid-phase exfoliation of graphite is gaining ground. Proteins consist of both hydrophilic and hydrophobic regions, and can therefore act as amphiphilic stabilizers for graphene and other 2D materials [11]. Their environmentally friendly nature, as well as their potential as catalysts, favor them over traditional chemical catalysts [12]. En route towards a more sustainable synthesis of graphene, researchers have used bovine serum albumin as well as other serum proteins as stabilizers for the production of graphene that increase its hydrophilicity and prevent restacking of the nanosheets [13].

Protein–graphene interaction can also create an ideal host for enzyme immobilization, and thus leverage graphene as an effective green immobilization matrix. Few studies have reported enzyme immobilization on bio-Graphene. For instance, horseradish peroxidase (HRP) was non-covalently immobilized on a few-layer bio-Graphene for biosensor development [14]. Comparing the performance of the prepared nanobiocatalyst with that of the free enzyme, the results were ideal in that the activity of the immobilized nanobiocatalyst was very close to that of the free enzyme, indicating that bio-Graphene can be a very promising green carrier for immobilization. In another study, glucose oxidase (GOx) and HRP were co-immobilized by interlocking on bio-Graphene-coated cellulose paper, to test if bio-Graphene can function as an electron shuttle, thereby optimizing the reaction efficiency between the enzyme and the substrate. The nanobiocatalyst presented increased activity and stability in the cascade reaction that was followed. [15].

In this study, we report the green synthesis of bio-Graphene (bG) through ultrasonication in water, using bovine serum albumin (BSA) as an exfoliating, stabilizing, and modifying agent. The prepared bG was employed as support for the development of a multi-enzymatic system, through the covalent co-immobilization of GOx, HRP, and *β*-glucosidase (bgl). Multi-enzymatic systems, when developed through simultaneous immobilization, are able to complete a reaction without the individual steps that are usually necessary in step-by-step procedures; they therefore result in shorter catalytic cycles, lower losses due to diffusion limitations, and the production of less by-products and intermediates. The accumulation of unwanted intermediates and by-products can be avoided because the intermediates act as substrates for the next reaction [16]. Based on this idea, a variety of enzymes act together, effectively overcoming limitations such as operating restrictions, and environmental and economic boundaries that free enzymes are not capable to overcome [17,18]. The bio-Graphene obtained as described was characterized by different microscopic and spectroscopic techniques; the multi-enzymatic nanobiocatalytic system prepared employing this bG was biochemically and morphologically characterized, and further applied in a three-step cascade reaction, involving, as the first step, the hydrolysis of *p*-Nitrophenyl-*β*-D-glucopyranoside (*p*NPG) and cellobiose. To the best of our knowledge, this is the first study investigating the potential of simultaneous covalent co-immobilization of three enzymes onto the surface of bio-Graphene and its further use in cascade reactions of biotechnological interest.

## 2. Materials and Methods

### 2.1. Materials

Bovine serum albumin (BSA, 98% Fraction V), graphite powder (<20 μm, synthetic), glucose oxidase from *Aspergillus niger* (GOx, 215 Units mg^−1^), horseradish peroxidase (HRP, 173 Units mg^−1^), 2,2′-azino-bis(3-ethylbenzothiazoline-6-sulfonic acid) diammonium salt (ABTS), glutaraldehyde solution (25 % *v*/*v*), *p*-nitrophenyl-*β*-D-glucopyranoside, (*p*NPG), and D(+)-glucose were purchased from Sigma-Aldrich (St. Louis, MO, USA). *β*-Glucosidase from *Thermotoga maritima* (bgl, 50 Units mg^−1^) was purchased from Megazyme (Chicago, IL, USA) and used without further purification. Polyoxyethylene sorbitan monoolate (TWEEN™-20) was purchased from Fluka (Charlotte, NC, USA), and D(+)-cellobiose was purchased from Alfa Aesar (Kandel, Germany). All the other chemicals and reagents were of analytical grade and procured from reliable sources. Milli-Q water was used for the preparation of all the buffers and solutions.

### 2.2. Synthesis of bio-Graphene

Bio-Graphene (bG) was prepared according to literature [19], with small modifications. Briefly, 100 mg of graphite were added to 20 mL of purified water. The sample was ultra-sonicated for 1 h (200 W, 10 kHz, pulser 50%) to partially exfoliate the graphitic sheets. Next, 5 mL of purified water were added to 100 mg BSA, then the sample was homogenized and transferred to the graphite suspension. The graphite–BSA mixture was allowed to stir for 1 h at room temperature, and then centrifuged at 500 rpm for 2 min to separate the non-exfoliated graphite. The supernatant was carefully separated from the non-exfoliated graphite precipitant. A second centrifugation at 8000 rpm for 10 min took place to remove the excess of BSA. 1 mL of acetone was added to dissolve the black powder (precipitate) and then the sample was transferred to a Petri dish and dried at 50 °C for 4 h. The production yield of bG was calculated using Equation (1):

(1) $$Production\;yield\;\left(\%\right)=\frac{mg\;of\;produced\;bG}{mg\;of\;graphite}\times 100$$

### 2.3. Characterization of bio-Graphene

Atomic Force Microscopy (AFM) images were collected in tapping mode with a Bruker Multimode 3D Nanoscope (Ted Pella Inc., Redding, CA, USA), using a microfabricated silicon cantilever type TAP-300G, with a tip radius <10 nm and a force constant of ~20–75 N m^−1^. Drop casting was used to deposit samples from aqueous solutions onto silicon wafers. The Si wafers (P/Bor, single-side polished, Si-Mat, Sigma-Aldrich, St. Louis, MO, USA) used in the AFM imaging were cleaned before use for 20 min in an ultrasonic bath (160 W) with water, acetone (≥99.5% Sigma-Aldrich, St. Louis, MO, USA), and ethanol (≥99.5% Sigma-Aldrich, St. Louis, MO, USA) [20]. Raman spectra were recorded using a Micro-Raman system RM 1000 (RENISHAW, Old Town, UK) with a laser excitation wavelength of 532 nm. The laser power was set at 0.5−1.0 mW, with a 1 μm focus spot to avoid photodecomposition of the samples. Spectra were collected three times per location for five locations per sample and averaged. Fourier-Transform Infrared (FTIR) spectra were recorded by a FTIR-8400 infrared spectrometer (Jasco, Tokyo, Japan) equipped with a deuterated triglycine sulfate (DTGS) detector. All spectra were recorded within 400–4000 cm^−1^ range, and an average of 32 scans was applied. Samples were prepared as KBr pellets containing ca. 1 wt% of the sample. Fluorescence measurements were recorded on a luminescence spectrofluorometer Jasco-8300 (Tokyo, Japan) equipped with a solid sample holder. Samples with a concentration of 1 mg mL^−1^ were drop-casted on silicon wafers (P/Bor, single side polished, Sigma-Aldrich, St. Louis, MO, USA) and left to dry. The fluorescence emission spectra were recorded in the region 300–400 nm after excitation at 280 nm, with a scan speed of 100 nm min^−1^. Slit widths with a nominal band pass of 5 nm were used for both excitation and emission ray. All emission measurements were taken at 25 °C and, for every scanned sample, a baseline was recorded and subtracted from the sample spectrum. The X-ray photoelectron spectroscopy (XPS) data were collected using a Surface Science Instruments SSX-100 ESCA instrument equipped with a monochromatic Al K_α_ X-ray source (hν = 1486.6 eV). The samples were dispersed in chloroform by sonication for 10 min, and a small drop of the suspension was left to dry in air on a homemade 150 nm thick gold film supported on mica [21]. During the measurement, the pressure was kept below 1.0 × 10^−9^ mbar in the analysis chamber; the electron take-off angle with respect to the surface normal was 37°. A flood gun was used during the XPS measurements to compensate for charging effects. The XPS data were acquired on a spot with a diameter of 1000 μm and the energy resolution was set to 1.26 eV for the spectra of the various core level regions, and to 1.67 eV for the survey scans. Binding energies (BE) are reported as ±0.1 eV when deduced from a fit and referenced to the C1*s* photoemission peak centered at a binding energy of 284.8 eV. All spectra were analyzed using the least squares fitting program Winspec (LISE laboratory, University of Namur, Belgium). Deconvolution of the spectra included a Shirley baseline subtraction, fitting with a minimum number of peaks consistent with the structure of the surface, taking into account the experimental resolution. The peak profile was taken as a convolution of Gaussian and Lorentzian functions. All measurements were carried out on two spots on freshly prepared samples to check for homogeneity. Scanning electron microscopy (SEM) images were recorded with an FEI-Philips FEG-XL30s microscope, and energy dispersive X-ray spectroscopy (EDS) was performed on the same instrument with the help of an EDAX Octane silicon drift detector with an accelerating voltage of 20 kV.

### 2.4. Preparation of the Tri-Enzymatic Nanobiocatalyst

The three enzymes bgl, GOx, and HRP (at a mass ratio 1:1:3) were attached on bG by a covalent immobilization procedure, involving the use of glutaraldehyde as the cross-linking agent. The mass ratio of the multi-enzymatic system was determined based on prior research, as well as the immobilization yield that each enzyme demonstrated when was individually immobilized (data not shown). Briefly, 10 mg of bG were dispersed in 17 mL of sodium phosphate buffer (100 mM, pH 6.0) and 0.3 mL of Tween-20, and the mixture was left in an ultrasonic bath for 15 min. Next, 15 mL of glutaraldehyde were added, and the mixture was incubated under stirring at 30 °C for 1 h. The activated bG was centrifuged at 4,000 rpm for 10 min, washed thrice with sodium phosphate buffer, and re-dispersed in 17 mL sodium phosphate buffer. In the next step, 3 mL of sodium phosphate buffer containing the three enzymes (mass ratio 1:1:3, bgl:GOx:HRP, respectively) were added to the activated bG and the mixture was incubated for 2 h at 30 °C with constant shaking at 150 rpm. The co-immobilized nanobiocatalyst was separated by centrifugation at 12,000 rpm for 10 min, washed thrice with sodium phosphate buffer, and left to dry under silica at 4 °C. Immobilization yield (%) of GOx and HRP was determined by recording protein spectra in the FAD (300–500 nm) and Soret region (400–450 nm), respectively, while the immobilization yield of bgl was calculated from the supernatant activity before and after the procedure. The activities of enzymes were expressed as activity recovery (%), which was determined as the percentage of the activity of each immobilized enzyme divided by the total initial activity of each enzyme.

### 2.5. Enzyme Assays

Enzymatic activity of free and co-immobilized bgl was determined by *p*NPG hydrolysis. For the activity assay, 0.12 or 100 μg mL^−1^ of free or co-immobilized enzyme, respectively, was mixed with 2.0 mM *p*NPG in 100 mM citrate phosphate buffer (100 mM, pH 6.5) at 50 °C. The reaction was followed by measuring the increase in absorbance of the released *p*-nitrophenol (*p*NP) at 410 nm for 5 min, using a UV/Vis spectrophotometer (Shimadzu, Tokyo, Japan). The molar extinction coefficient (ε = 18.3 mM^−1^ cm^−1^) of the produced *p*NP at 410 nm was used to convert the absorbance values to *p*NP concentration [22]. Enzymatic activity was estimated as the amount (μmoles) of *p*NP produced per minute.

For determining the HRP activity, the enzymatic assay was based on the oxidation of ABTS to ABTS^+^ in the presence of hydrogen peroxide. For the assay, 3 mM ABTS, 0.1 mM hydrogen peroxide, and 0.4 μg mL^−1^ of free or 100 μg mL^−1^ of co-immobilized enzyme were mixed in sodium phosphate buffer (100 mM, pH 7.0). The reaction was followed for a total of 5 min by measuring the increase in absorbance of oxidized ABTS at 405 nm, using a UV/Vis spectrophotometer (Shimadzu, Tokyo, Japan). The molar extinction coefficient (ε = 36.8 mM^−1^ cm^−1^) of oxidized ABTS at 405 nm was used to convert the absorbance values to ABTS^+^ concentration [23]. Enzymatic activity was determined as the amount (μmoles) of ABTS transformed per minute.

The determination of the GOx activity is based on the coupling reaction of GOx with HRP. D-glucose is converted by GOx to D-glucono-S-lactone and hydrogen peroxide, and in the next step, HRP converts ABTS to ABTS^+^ via hydrogen peroxide. Briefly, 250 mM of glucose solution, 0.4 mM ABTS, 11 μg mL^−1^ HRP, and 0.5 μg mL^−1^ of free or 50 μg mL^−1^ of co-immobilized GOx, were added in phosphate buffer (50 mM, pH 7.0). The reaction was followed for 5 min by measuring the increase in absorbance of oxidized ABTS at 405 nm using a UV/Vis spectrophotometer (Shimadzu, Tokyo, Japan). Enzymatic activity was determined as the amount (μmoles) of ABTS transformed per min.

In the case of the three co-immobilized enzymes on bG, the activity of the nanobiocatalyst was monitored by using a combination of a) *p*NPG and ABTS, and b) cellobiose and ABTS, as the initial substrates. *p*NPG or cellobiose was hydrolyzed to D-glucose by bgl, which was oxidized by GOx to gluconic acid and H_2_O_2_, followed by the reduction of H_2_O_2_ to H_2_O by HRP via ABTS oxidation. For the enzyme assay, 1 mg mL^−1^ of the co-immobilized biocatalyst was added in phosphate buffer (100 mM, pH 6.0). The reaction was initialized with the addition of 3 mM ABTS and 0.5 mM *p*NPG or cellobiose, for the two cases studied. Absorbance spectra in the region 400–800 nm were recorded at a UV/Vis spectrophotometer (Shimadzu, Tokyo, Japan) for different time intervals, at 25, 30, 37, and 50 °C.

### 2.6. Determination of Michaelis–Menten Kinetic Parameters

The apparent kinetic parameters (*K*_m_, *V*_max_) of free and co-immobilized forms of bgl, GOx, and HRP were estimated by determining the initial reaction rates for each enzyme form at 37 °C (for GOx and HRP) and at 50 °C (for bgl), using different concentrations of *p*NPG (0.05–25 mM), glucose (0.25–25 mM), and H_2_O_2_ (0.025–0.3 mM). For HRP assays, the reactions were performed in the presence of 3 mM ABTS, whereas for GOx assays, the reactions were performed with 2 mM ABTS.

### 2.7. Thermal Stability Studies

The thermal stability of GOx, bgl, and HRP in free and co-immobilized form was investigated at 37–60 °C. Samples were taken at regular time intervals and the remaining enzymatic activity was measured as described above.

### 2.8. Reusability Studies

The reusability studies of the co-immobilized enzymes, in terms of activity against their respected substrates, were performed at 30 °C for a specified duration. Activity was determined using the appropriate substrates (glucose, *p*NPG, and H_2_O_2_). The nanobiocatalysts were separated by centrifugation (12,000 rpm) after each reaction cycle, rinsed completely with buffer (sodium phosphate 100 mM, pH 7.0 for GOx and HRP, and citrate phosphate 100 mM, pH 6.5 for bgl), and re-suspended in a fresh substrate solution to begin a new reaction cycle. The assay was performed for 4 successive catalytic cycles. The relative activity of each enzyme (%) was defined as the ratio of the remaining activity to the activity of the first cycle.

The reusability study of the three-enzymatic cascade reaction was performed at 30 °C for *p*NPG hydrolysis, and at 50 °C for cellobiose hydrolysis, for a specified reaction duration (2.5 and 4.5 h, respectively). Activity was determined using the appropriate substrates (*p*NPG or cellobiose and ABTS). Absorbance spectra were recorded in the region 400–800 nm as previously described. The multi-enzymatic nanobiocatalyst was separated by centrifugation (12,000 rpm) after each reaction cycle, rinsed completely with buffer (sodium phosphate 100 mM, pH 6.0), and re-suspended in a fresh substrate solution to begin a new reaction cycle. The assay was performed for up to 4 successive catalytic cycles, and the relative activity (%) was defined as the ratio of the remaining activity to the activity of the first cycle.

## 3. Results and Discussion

### 3.1. Effect of the Graphite and Bovine Serum Albumin Concentration on the Production of bio-Graphene

**Figure 1:**
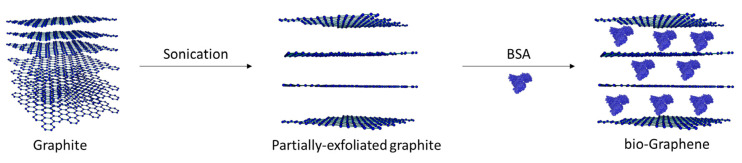
Schematic presentation of the production of bio-Graphene.

In the present work, an easy and environmentally friendly method for producing bio-functionalized few-layer bio-Graphene is reported (Figure 1). Biosynthesis of bG was carried out in water, using BSA as the exfoliation agent and stabilizer of the graphitic sheets. The first step included the partial exfoliation of graphitic sheets by the application of ultrasonic waves. In the next step, BSA was added to the partially-exfoliated nanomaterial to stabilize the layers by physical adsorption on the graphitic sheets [13]. The selected pH value (pH = 7.0) for the synthesis of bG was based on the negative charge of BSA (isoelectric point = 5.3) in the reaction medium, as it has been reported that the production of graphene is strongly correlated to the isoelectric point of proteins, and more specifically, to the distribution of negative charges [24].

**Figure 2:**
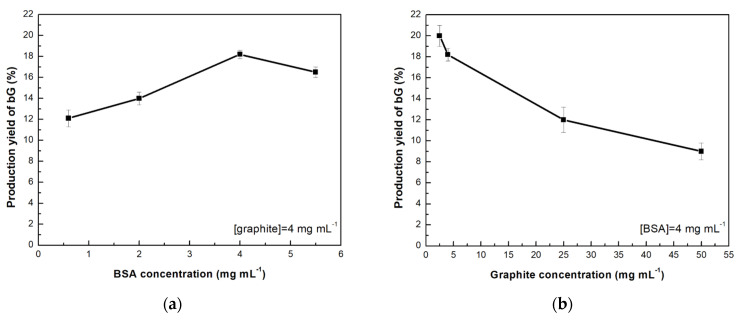
Production yield (%) of bio-Graphene as a function of (**a**) bovine serum albumin, and (**b**) graphite concentration.

The production yield of bG was determined by testing different BSA concentrations (0.6–5.5 mg mL^−1^), using a fixed concentration of 4 mg mL^−1^ graphite, which corresponds to 100 mg of initial material. Graphite is abundant and cheap, thus the selection of this initial amount was based on the potential application in large-scale productions. The results are presented in Figure 2a, showing that the increase of BSA concentration from 0.6 to 4 mg mL^−1^ resulted in high production yield of bG, while above concentrations of 4 mg mL^−1^, the production efficiency started to decrease. A similar effect of the initial BSA concentration on the production rate of bG was previously reported, where in that case too, above a certain protein concentration, no significant increase in the exfoliation yield was detected [24]. Following this observation, the effect of graphite concentration was also investigated, using a fixed BSA concentration of 4 mg mL^−1^ (Figure 2b). It can be observed that the increase of graphite concentration results in a decrease of bG production yield. This could be attributed to the limited interactions between the material and the protein, due to excess of graphite, indicating that the BSA:graphite ratio plays a critical role during the exfoliation procedure. For the next studies, a mass ratio 1:1 (BSA:graphite) was chosen, as this combination provided the highest production yield of bG.

### 3.2. Microscopic and Spectroscopic Characterization of bio-Graphene

#### 3.2.1. Microscopic Characterization

**Figure 3:**
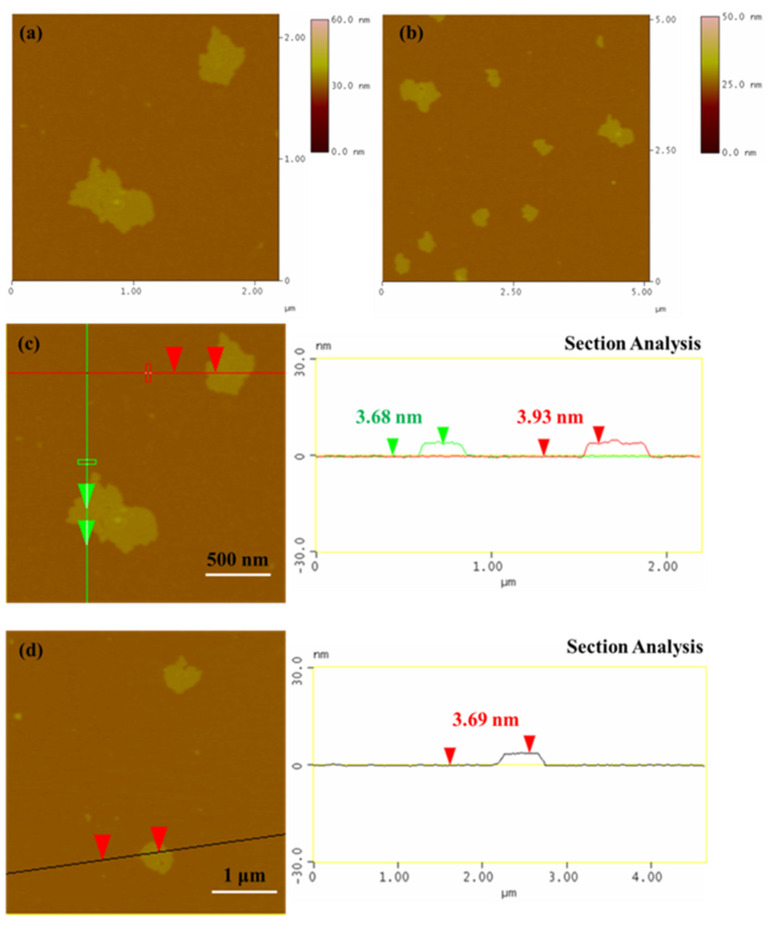
Morphology of the bio-Graphene flakes: (**a**,**b**) AFM images, and (**c**,**d**) height profile analysis along the lines marked in the images.

The protein penetrates in the graphite interlayer space by electrostatic, hydrophobic, and π–π stacking interactions and thereby causes exfoliation into flakes [13]. AFM was used to measure the number of layers of the bG flakes drop-casted from the solution, to evaluate whether the exfoliation process was effective (Figure 3). The AFM images confirm the successful synthesis of bG, as the height AFM images (Figure 3a,b) demonstrate the presence of bG flakes, which are homogeneous and without spots on their surface. Their lateral dimensions range from 0.5–1 μm. The average thickness of the flakes, as shown by the height profile images (Figure 3c,d), ranges from 3.5–4.0 nm, which indicates that two to four monoatomic bio-Graphene sheets are stacked together in the flakes. In a previous work, AFM confirmed the presence of few-layer graphene flakes with a thickness of 6.7 ± 4.6 nm after sonication of graphite in aqueous BSA solution [13].

**Figure 4:**
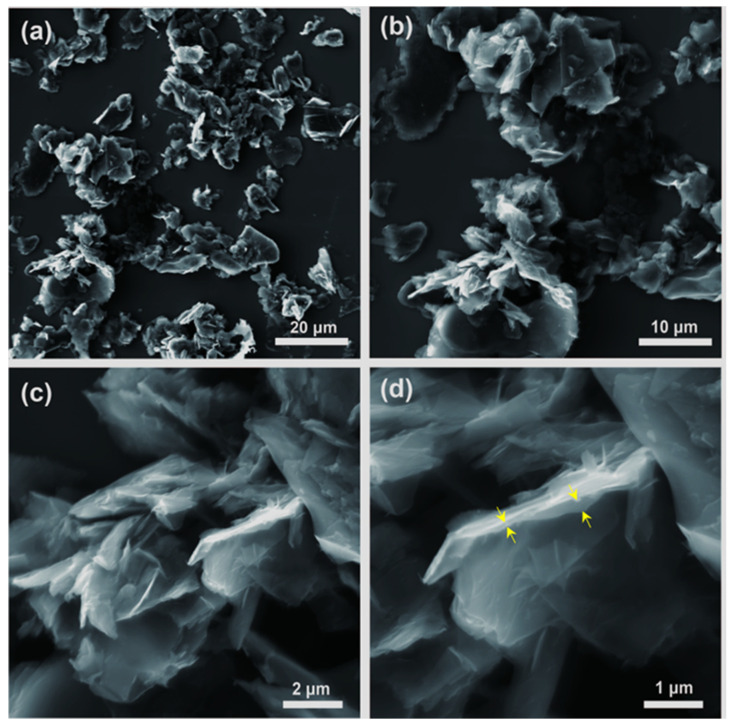
(**a**–**d**) SEM images of bio-Graphene (yellow arrows show the interlayer space of graphene flakes).

SEM images of bG at different length scales are presented in Figure 4: few-layer graphene flakes can here clearly be recognized in agreement to what was deduced from the height of the flakes measured by AFM. The enlarged cross-sectional view (Figure 4d), yellow arrows) further confirms that BSA must be intercalated between graphene layers, leading to a more loosely stacked pillared structure. Although the flakes aggregate during the drying process, some isolated flakes can also be distinguished, and the size of the graphene flakes reaches up to several micrometers.

#### 3.2.2. Spectroscopic Characterization

**Figure 5:**
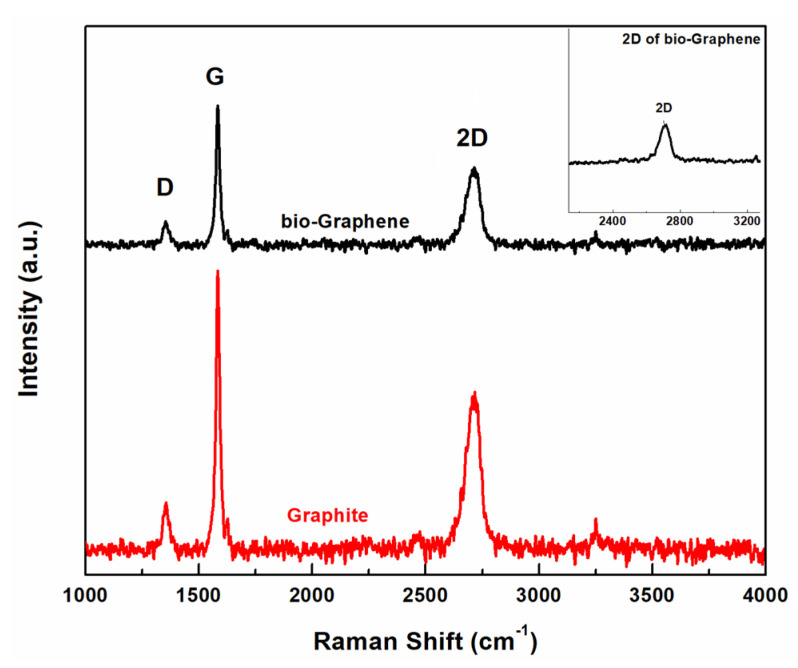
Raman spectra of graphite and bio-Graphene.

Raman spectroscopy was used to obtain information for the produced bG. The Raman spectrum of graphite showed the peaks of D, G, and 2D bands at ~1347, ~1579, and ~2710 cm^−1^, respectively, which are the characteristics for pristine graphite (Figure 5). In the case of bG, the bands D, G, and 2D bands were observed at ~1350, ~1582, and ~2708 cm^−1^, respectively, presenting differences both in intensity and shape, compared to graphite. A slight increase in the I_D_/I_G_ ratio from 0.13 (graphite) to 0.2 (bG) was noted after exfoliation; although this difference is not significant, it could be ascribed to the electrostatic interactions between graphene sheets and BSA and to the presence of aromatic residues on the surface of bG [13]. Moreover, the formation of more edges that were created during exfoliation addresses more defects in graphene sheets [25,26].

The intensity ratio I_2D_/I_G_ is a very common value for determining the exfoliation of graphene. When the value of this ratio I_2D_/I_G_ is equal to 2.0, a perfect graphene monolayer is expected to be formed, while when the ratio is equal to 1.0, it is indicative of the presence of a bilayer. In our case, the ratio was calculated at 0.45 for graphite and 0.64 for bG, indicating the successful exfoliation of graphite in few-layered graphene [25]. Finally, the 2D band of bG presented in the Raman spectrum was more symmetrical than the one of the graphite (the 2D peak of bG is presented in the enlarged inset), demonstrating the lack of stacking of the graphene layers, and thus the production of exfoliated graphene.

**Figure 6:**
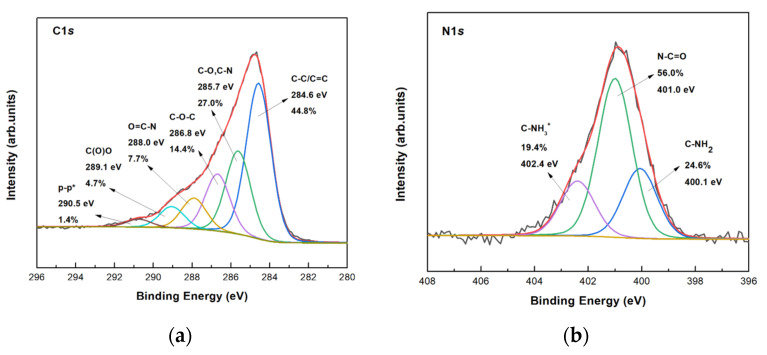
XPS spectra of the (**a**) C1*s*, and (**b**) N1*s* core level regions of bio-Graphene.

XPS was employed to identify the chemical groups of bG after exfoliation by BSA in aqueous solution. The wide scan survey spectrum of bG is presented in Figure S1, where the spectroscopic signature of C, N, and O can be observed. No other atoms were observed during the exfoliation, as expected when the solvent involved in the exfoliation procedure is water. The XPS spectra of the C1*s* and N1*s* core level regions of bG are presented in Figure 6. The C1*s* line was deconvoluted with six components as presented in Figure 6a. The main peak at a binding energy (BE) of 284.6 eV can be assigned to C–C/C=C bonds of the graphene layers and in the carbon backbone of BSA, while the component centered at 285.7 eV can be attributed to C–N (C–NH_2_) and C–O bonds, and the one at 288.0 eV to the peptidic carbons (O=C–N) of albumin. The shake-up satellites at about 6 eV from the main C–C photoemission peak are generated by π–π* transitions happening together with the emission of the photoelectron, mainly on the phenyl groups of BSA and graphene. The detailed XPS spectrum of the N1*s* core level region in Figure 6b contains contributions from NH_2_ (BE = 400.1 eV), peptidic nitrogen (BE = 401.0 eV), and NH^3+^ (BE = 402.4 eV.) [27].

**Figure 7:**
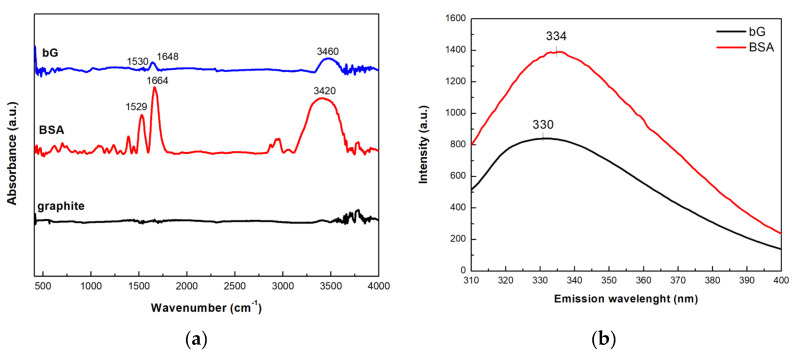
(**a**) FTIR and (**b**) fluorescence spectra of graphite, BSA, and bio-Graphene.

The FTIR and fluorescence spectra of graphite, BSA, and bG were recorded to confirm the biofunctionalization of bG with BSA. As shown in Figure 7a, the FTIR spectrum of pristine graphite lacks vibrational features because there are no functional groups on its surface. In the FTIR BSA spectrum, the band at 1664 cm^−1^ corresponds to the amide I region, arising from the C=O stretching vibrations of the protein peptide chain [28,29]. The peak near 1529 cm^−1^ can be ascribed to the N–H bending vibrations of the –NH_2_ groups of the protein, while the peaks in the region 1300–1450 cm^−1^ can be attributed to the amide III band [30]. Finally, the band at 3420 cm^−1^ arises from O–H stretching vibrations [31]. The presence of most intense of these bands in the bG spectrum (1648 and 1530 cm^−1^) confirm the successful intercalation of graphite with the protein molecules.

The successful functionalization of bG with BSA was also confirmed by fluorescence spectroscopy. BSA, due to its aromatic amino acids, exhibits a fluorescence emission maximum (λ_em_) at 334 nm, after excitation at 280 nm (Figure 7b). In the case of bG, a peak at 330 nm was observed pointing to the presence of BSA, since graphite does not emit in that region when excited at the aforementioned wavelength. The observed slight red-shift of the maximum λ_em_ of BSA (300 nm) suggested changes in the microenvironment near the aromatic amino acids of the protein, due to interactions between graphite and BSA.

### 3.3. Bio-Graphene as Support for a Tri-Enzymatic Co-Immobilized System

#### 3.3.1. Preparation of the Tri-Enzymatic Biocatalyst

The prepared bG was used as support for the simultaneous co-immobilization of three enzymes, namely GOx, HRP, and bgl, to develop a multi-enzymatic system able to catalyze cascade reactions. The covalent immobilization was achieved by exploiting the free amine groups present on the surface of BSA, which were used to cross-link the free amine groups (mostly derived from lysine residues) on the surface of the enzymes, through the formation of a Schiff’s base [32,33]. Glutaraldehyde was applied as the cross-linking agent. The immobilization yield (%) and activity of recovery (%) of each co-immobilized enzyme are presented in Table 1. The three enzymes exhibited different immobilization yields in the final tri-enzymatic system, a result that could be correlated to the size of each protein molecule. GOx and bgl can be found as dimers with approximately 160 kDa [34] and 95 kDa [35] total molecular weight, respectively, while HRP is a smaller molecule with a molecular weight of ~44 kDa [36]. Thus, it is possible that bgl and GOx prevail during the immobilization procedure, occupying most of the surface of bG, while the arrangement of HRP is restricted. This assumption could also explain the activity recovery of the co-immobilized enzymes. The activity of GOx and bgl was determined as 66.5 and 90%, respectively, indicating that these enzymes can preserve their catalytic activity upon immobilization. On the contrary, HRP recovered half of its activity after co-immobilization, possibly due to either conformational changes of the HRP molecule, or to the arrangement of HRP within the complex, restricting the diffusion of the substrates towards its active sites [37].

#### 3.3.2. Characterization of the Co-Immobilized Enzymes on bio-Graphene

**Figure 8:**
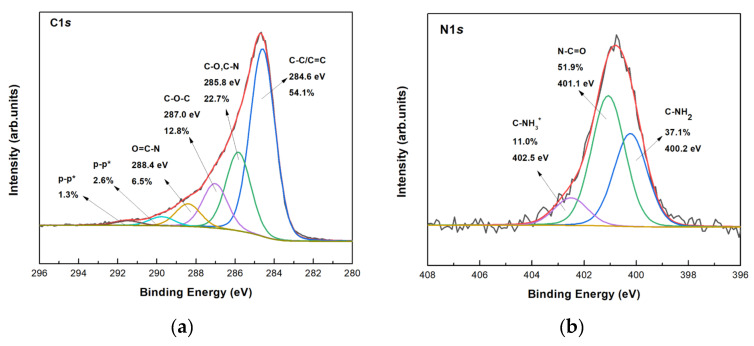
XPS spectra of the (**a**) C1*s* and (**b**) N1*s* core level regions of the tri-enzymatic system immobilized on bio-Graphene.

After the co-immobilization of the three enzymes in the final layered biomaterial, XPS was used to confirm the presence of the enzymes on bG and try to learn how they interact with it. Firstly, an increase of the relative spectral intensity of the C–C/C=C component in the C1*s* line was observed and this demonstrated the successful incorporation of the enzymes (Figure 8a). The high amount of amine end groups of the enzymes is mirrored in an increase of the spectral intensity of the component due to C–NH_2_ bonds in the N1*s* line (Figure 8b), which attests to the successful immobilization on bG. The signal of amine and amidic bonds are significantly stronger than C=N bonds correlated to the Schiff’s base formation during the covalent immobilization, so the C=N bond signal cannot be resolved in the N1*s* spectrum.

**Figure 9:**
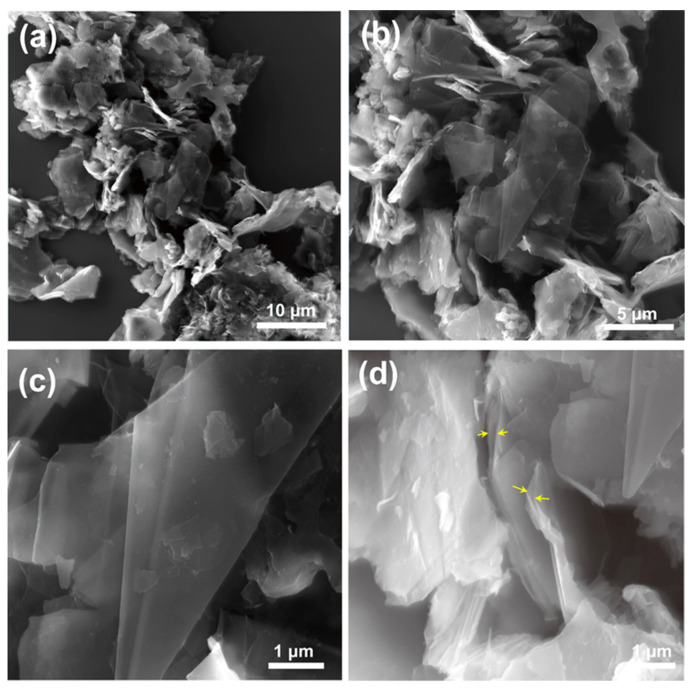
(**a**–**d**) SEM images of the tri-enzymatic system on bio-Graphene (yellow arrows shows the interlayer space of graphene flakes).

SEM was used to visualize the morphology of the tri-enzymatic system on bG. After the enzyme interaction, one still sees a layered structure and the graphene flakes have the same lateral dimensions as before grafting the enzymes (Figure 9). However, the final nanosystem seems better exfoliated than the bio-Graphene presented in Figure 4, as graphene monolayers are observed more frequently. In addition, the cross-section view of graphene flakes in Figure 9d shows a larger interlayer spacing and better exfoliation. This is perhaps because during the immobilization procedure, the enzymes work synergistically to further delaminate the graphene layers. EDX spectra and elemental mapping of the tri-enzymatic system showed the characteristic N, O signals, and both of these are homogeneously distributed over the flakes (Figures S2 and S3). This demonstrates that both functionalizing agents cover the substrate uniformly.

#### 3.3.3. Kinetic Studies of the Co-Immobilized Enzymes on bio-Graphene

The apparent kinetic constants (*K*_M_ and *V*_max_) of the free and the co-immobilized form of each enzyme were calculated by determining the initial reaction rates at varying substrate concentrations. As shown in Table 2, in all cases the apparent *K*_M_ values of the co-immobilized form of the enzyme were higher than that of the free enzyme. This increase in *K*_M_ values suggests that the affinity of the enzymes towards their respective substrates decreases after immobilization on bG. Co-immobilization of enzymes on bG may result in conformational changes of the protein molecules, limiting their mobility, and hence decreasing substrate diffusion to their active sites [38,39]. These results agree with previous studies [38,40,41,42]. Moreover, following immobilization, the apparent *V*_max_ values were all significantly reduced, indicating restricted enzymatic reaction rates towards their substrates. This is most likely caused by a decrease in the available active sites, due to the immobilization process, as a result of the biocatalyst’s rigidification when it is restricted by the glutaraldehyde cross-linker [39]. Furthermore, the immobilization yield of each enzyme seems to play an important role for their co-localization in the complex; if one or two of the enzymes prevail over the others, only a reduced amount of the minority enzyme will be immobilized due to the limitation of the area of the support, leading to lower catalytic activity [43].

#### 3.3.4. Thermal Stability of the Co-Immobilized Enzymes on bio-Graphene

The thermal stability of enzymes is considered a significant indicator for their application in large-scale procedures [44]. The thermal stability of the free and the co-immobilized form of the enzymes bgl, GOx, and HRP was investigated after incubation in sodium phosphate buffer at different temperatures (37–60 °C). As can be seen in Figure S4, both free and co-immobilized GOx remained stable even after 24 h incubation at 37 °C, while in the case of HRP, the co-immobilized biocatalyst presented higher residual activity than the free form of the enzyme. Stability studies at 37 °C were not conducted for bgl, as this enzyme is highly thermostable [38]. Similar results were obtained after incubation at 50 °C (Figure S5). In the case of HRP, a decrease in the catalytic activity of the enzyme was observed, which was higher for free HRP than for the co-immobilized enzyme, indicating that immobilization improves the stability of the protein. Moreover, bgl in both forms preserved its total catalytic activity, as expected.

**Figure 10:**
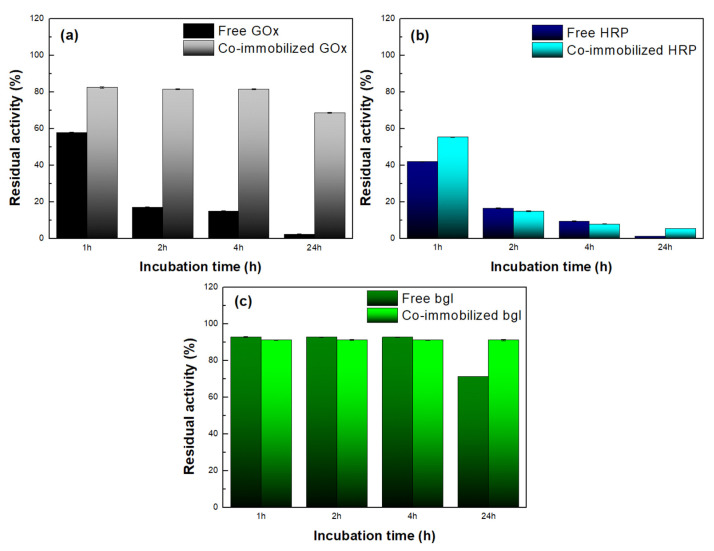
Residual activity of (**a**) free and co-immobilized GOx, (**b**) free and co-immobilized HRP, and (**c**) free and co-immobilized bgl after incubation at 60 °C.

The most pronounced results concern the residual activity of the nanobiocatalysts after incubation at 60 °C (Figure 10). Figure 10a shows that free GOx lost more than 50 % of its catalytic activity after 1 h of incubation, while after 24 h, it was almost deactivated. On the contrary, co-immobilized GOx preserved up to 70 % of its initial activity after 24 h of incubation, indicating that immobilization on bG results in a more stable biocatalyst. The formation of covalent bonds between the enzyme and bG offers thermal protection, and thus decreases the rate of enzymatic denaturation at high temperatures, as has also been reported in other works [44]. A similar effect was also observed for bgl (Figure 10c). Both forms of the enzyme were stable for up to 4 h of incubation at 60 °C, while after 24 h, the co-immobilized bgl preserved its catalytic activity to a higher extent than the free enzyme. On the contrary, the remaining activity of both free and co-immobilized HRP decreased with incubation time (Figure 10b), but after 24 h, the co-immobilized HRP showed a higher residual activity than the free enzyme and hence appeared to be more stable. Overall, the results showed that bG enhances the stability of all the co-immobilized enzymes compared to their respective free forms. This stabilizing effect could derive from the morphology of the nanomaterial; the flexible layered structure of bG protects the conformation of proteins against heat denaturation, as is already known for graphene-based nanomaterials [45]. Moreover, the enhanced stability of the immobilized enzymes on bG can be correlated to the presence of BSA within the graphene flakes. BSA has the ability to separate the enzyme molecules from each other, inhibiting, in this manner, enzyme aggregation, and thus promoting enzyme stability [15].

#### 3.3.5. Reusability of Co-Immobilized Enzymes on bio-Graphene

**Figure 11:**
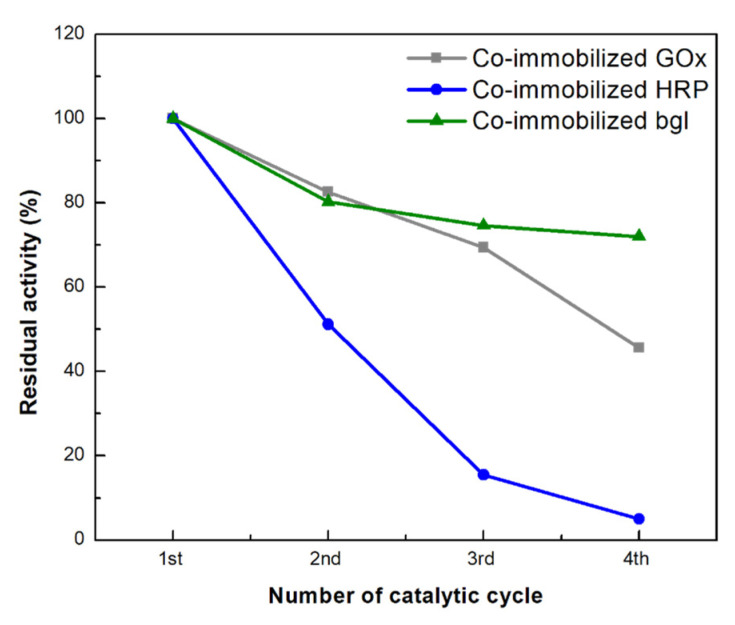
Reusability of the co-immobilized enzymes on bG for their respective substrates: residual activity as a function of number of cycles. The activity at the first catalytic cycle was set at 100% (error bars are too small to be visible).

In industrial applications, it is crucial that enzymes can be effectively recovered and re-applied in successive catalytic cycles, as this decreases the cost of the processes. Therefore, the reusability of the co-immobilized enzymes towards their respective substrates was investigated for four catalytic cycles (Figure 11). The residual activity of each co-immobilized enzyme gradually diminished during four consecutive cycles. More specifically, co-immobilized bgl was found to be the most stable enzyme in the tri-enzymatic system since it preserved up to 72 % of its initial catalytic performance in the fourth successive reaction cycle. Co-immobilized GOx retained around 45 % of its initial activity in the fourth biocatalytic cycle, while co-immobilized HRP retained only 16 % of its initial activity in the third catalytic cycle and was almost deactivated when exposed to the fourth batch. The decrease in enzyme activity could arise either from inhibition phenomena by the forming products, or from structural and/or mechanical deformations of the nanobiocatalysts [39]. Similar results have also been reported for the co-immobilization of cellulase and GOx on graphene oxide, where co-immobilized GOx retained 64.14 % after seven catalytic cycles [42], as well as for the co-immobilization of cellulase, bgl, GOx, and HRP onto amino-functionalized magnetic nanoparticles, where GOx and HRP preserved 55 and 22 % of its initial activity, respectively, after five catalytic cycles [38].

#### 3.3.6. Application of the Immobilized Tri-Enzymatic Biocatalyst on bio-Graphene in Cascade Reactions

The ability of the tri-enzymatic nanobiocatalyst to conduct a three-step chain reaction was studied for two catalytic reactions, involving either the initial *p*NPG or cellobiose hydrolysis (Figure S6). In these cascade reactions, in the first step, bgl hydrolyzes *p*NPG (or cellobiose) into simpler monosaccharides, such as glucose. The next step of this cascade reaction involves the GOx-mediated oxidation of glucose to gluconic acid and H_2_O_2_. Lastly, the produced H_2_O_2_ acts as a substrate for HRP, which, along with the chromogenic substrate ABTS, performs the final oxidizing step. The reactions are followed by recording the intensity of the absorption peak at 745 nm, which corresponds to monitoring the formation of the final product (ABTS^+^) and thus reveals the success of the chain reaction.

**Figure 12:**
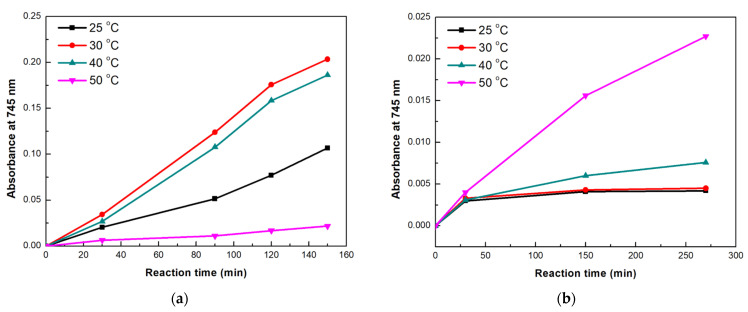
Time-dependent increase in absorption at 745 nm for cascade reactions conducted at different temperatures, using as initial substrate (**a**) *p*NPG and (**b**) cellobiose. (Standard errors were <0.05 in all cases).

The first cascade reaction catalyzed by the tri-enzymatic system was based on hydrolysis of *p*NPG, as the initial step. The reaction was conducted at different temperatures and the results are presented in Figure 12a, while the corresponding spectra can be found in Figure S7. The reaction rates of the tri-enzymatic immobilized system were up to 50-fold lower than those of the tri-enzymatic free system, regardless the reaction temperatures. This result was expected as the activity of the immobilized enzymes was decreased upon immobilization, as already shown in Table 2. As can be seen, when temperature was raised from 25 to 40 °C, the cascade reaction proceeded at a higher rate, as indicated by the intensity increase of the 745 nm-absorption band. Similar results were observed in a previous study, where multi-enzyme films, containing bgl, GOx, and HRP immobilized on polymers, were able to catalyze the same three-step cascade reaction within 180 min, at 25 and 37 °C [46]. Higher temperatures were expected to increase the cascade reaction rate, since the first step of the reaction that is catalyzed by bgl would be favored. Based on the literature, glucosidases from *T. maritima* are effective catalysts towards glycosidic bonds hydrolysis, exhibiting high conversions rates under elevated temperatures [41]. However, at 50 °C, a significant decrease in the tri-enzymatic activity was observed. This result could be correlated to the low stability that HRP presented at 50 °C (Figure S5b); a possibly inactivated HRP in combination with the excess of H_2_O_2_ generated by GOx could explain the decreased enzymatic performance of the nanobiocatalyst [47].

We also tested the tri-enzymatic nanobiocatalyst for the cascade where cellobiose hydrolysis is the initial step; the results for different reaction temperatures are shown in Figure 12b. The highest catalytic activity was observed at 50 °C, which is known to be the optimal operating temperature for *β*-glucosidases towards cellobiose hydrolysis [48]. It is interesting to note that contrary to *p*NPG hydrolysis, in this cascade reaction, the tri-enzymatic nanobiocatalytic system is effective at 50 °C. Cellobiose hydrolysis is a more complicated reaction than *p*NPG hydrolysis. The first step of the cascade reaction (hydrolysis of cellobiose to glucose by bgl) is slower in this case, which in turn affects the activity of GOx, and thus delays the production of high concentrations of H_2_O_2_, leaving HRP able to catalyze more efficiently the oxidation of ABTS.

**Figure 13:**
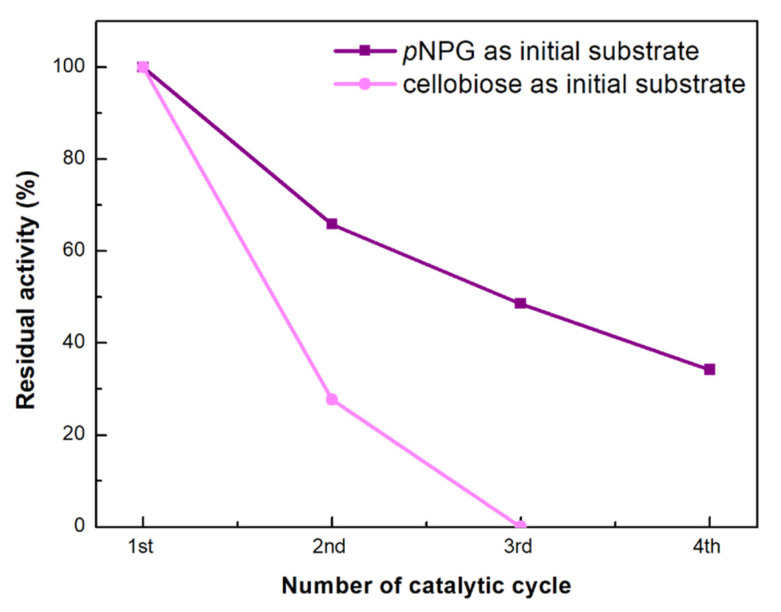
Reusability of the tri-enzymatic nanobiocatalytic system for the cascade reaction using as initial substrate *p*NPG and cellobiose, at 30 and 50 °C: residual activities during four consecutive cycles. The activity at the first catalytic cycle was set at 100% (error bars are too small to be visible).

Finally, the reusability of the tri-enzymatic nanobiocatalytic system was investigated towards the cascade reactions having as initial substrates *p*NPG and cellobiose, and the results are presented in Figure 13. The residual activity of the tri-enzymatic nanobiocatalyst for *p*NPG hydrolysis decreased after each catalytic cycle (each batch was left to react for 2.5 h) but the multi-enzymatic system preserved 34% of its initial activity in the fourth successive cycle. This reduction could be caused by a variety of factors such as product inhibition or mechanical damage to the nanobiocatalyst during recycling [39,44]. Regarding the cascade reaction involving cellobiose as the initial substrate, the activity of the nanobiocatalyst significantly decreased already in the second reaction cycle (28%), and the catalyst was no longer active in the third cycle (each batch reaction lasted for 4.5 h). In this case, the decrease in activity could be associated with HRP deactivation, due its low thermal stability, already discussed above.

## 4. Conclusions

In the present study, an environmentally friendly method was used to produce high-quality few-layer graphene sheets. BSA was used as an exfoliating and stabilizing agent, as well as a surface modifier for graphitic sheets, providing bG with different functional groups. Microscopic and spectroscopic techniques confirmed the presence of 3–4-layer graphene and informed on the quality of the nanomaterial. For the first time, the produced bG was used as a nanosupport for the simultaneous covalent co-immobilization of bgl, GOx, and HRP to develop a multi-enzymatic biocatalytic system. Although the co-immobilization procedure led to a loss in enzymatic activities, the developed tri-enzymatic nanobiocatalyst exhibited enhanced thermal stability, compared to the free forms of the enzymes, as well as good reusability. Furthermore, the developed nanobiocatalytic system was effectively applied in multi-step cascade reactions using two different glucosides as the initial substrate. The results pronounce the potential use of such multi-enzymatic systems to overcome economic and operating boundaries that the use of free enzymes is not capable of overcoming, such as lower losses due to diffusion limitations, and the ability to recover and reuse the biocatalyst for successive reaction cycles. The green synthesis of bG, together with the implementation of eco-friendly biocatalysts, such as enzymes, offer exciting possibilities, paving the way to the development of more sustainable catalysts for a variety of biological applications.

## Figures and Tables

**Table 1:** Immobilization yield (%) and activity recovery (%) of GOx, HRP, and bgl co-immobilized on bG.

Enzyme	Immobilization Yield (%)	Activity Recovery (%)
Co-immobilized GOx	80.5 ± 2.3	66.5 ± 3.7
Co-immobilized HRP	51.0 ± 3.2	50.0 ± 2.3
Co-immobilized bgl	98.5 ± 5.6	90.0 ± 2.8

**Table 2:** Apparent kinetic constants of free, individually immobilized, and co-immobilized form of GOx, HRP, and bgl.

Biocatalyst	Apparent *V*_max_ (μΜ min^−1^)	Apparent *K*_M_ (mM)
Free GOx	13.81 ± 0.48	2.79 ± 0.26
Co-immobilized GOx	3.33 ± 0.21	12.57 ± 2.31
Free HRP	2.93 ± 0.06	3.90 ± 0.32
Co-immobilized HRP	0.23 ± 0.05	9.90 ± 2.00
Free bgl	7.27 ± 0.50	0.22 ± 0.08
Co-immobilized bgl	2.88 ± 0.20	0.50 ± 0.14

## Data Availability

Not applicable.

## Supplementary Materials

The following supporting information can be downloaded at: https://www.mdpi.com/article/10.3390/nano13010127/s1, Figure S1: XPS survey spectrum of bio-Graphene; Figure S2: EDX spectrum of the tri-enzymatic system immobilized on bio-Graphene; Figure S3: SEM image (top left panel), and EDX mapping of C (middle left panel), O (middle right panel), N (bottom left panel) and P (bottom right panel) as well as overlay (top right panel) of the maps of a flake of tri-enzymatic system immobilized on bio-Graphene; Figure S4: Stability of free and co-immobilized GOx and HRP on bio-Graphene: residual activity after incubation at 37 °C for different times; Figure S5: Stability of free and co-immobilized GOx, HRP, and bgl on bio-Graphene: residual activity after incubation at 50 °C for different times; Figure S6: Schematic presentation of the cascade reaction of *p*NPG and cellobiose hydrolysis to ABTS^+^, catalyzed by the tri-enzymatic nanobiocatalyst; Figure S7: Time-dependent increase in absorption intensity at 745 nm for the cascade reaction of *p*NPG hydrolysis to ABTS^+^, catalyzed by the tri-enzymatic nanobiocatalyst at (a) 25 °C, (b) 30 °C, (c) 40 °C, and (d) 50 °C.
