# Supplementary material for: Development of a Multi-Enzymatic Biocatalytic System through Immobilization on High Quality Few-Layer bio-Graphene
Source: Nanomaterials (Basel). 2022 Dec 26;13(1):127. doi: 10.3390/nano13010127 (PMC9824680; doi:10.3390/nano13010127)
Supplement: Supplementary file 1 [file nanomaterials-13-00127-s001.zip › nanomaterials-2116046-supplementary.pdf]

SUPPLEMENTARY MATERIAL

Development of a Multi-enzymatic Biocatalytic System through Immobilization on High Quality Few-Layer bio-Graphene

Christina Alatzoglou [1], Michaela Patila [1*], Archontoula Giannakopoulou [1], Konstantinos Spyrou [2], Feng Yan [3], Wenjian Li [4], Nikolaos Chalmpes [2], Angeliki C. Polydera [1], Petra Rudolf [3], Dimitrios Gournis [2*] and Haralambos Stamatis [1*]

1 Laboratory of Biotechnology, Department of Biological Applications and Technology, University of Ioannina, 45110 Ioannina, Greece; ch.alatzoglou@uoi.gr (C.A.); arxontoula.gian@gmail.com (A.G.); apolyder@uoi.gr (A.C.P.)
2 Department of Materials Science & Engineering, University of Ioannina, 45110 Ioannina, Greece; konstantinos.spyrou1@gmail.com (K.S.); chalmpesnikos@gmail.com (N.C.)
3 Zernike Institute for Advanced Materials, University of Groningen, Nijenborgh 4, Groningen 9747AG, The Netherlands; f.yan.rug@gmail.com (F.Y.); p.rudolf@rug.nl (P.R.)
4 Engineering and Technology Institute Groningen, University of Groningen, Nijenborgh 4, 9747AG Groningen, the Netherlands; wenjian.li@rug.nl
* Correspondence: hstamati@uoi.gr (H.S.); dgourni@uoi.gr (D.G.); pstm10345@uoi.gr (M.P.)

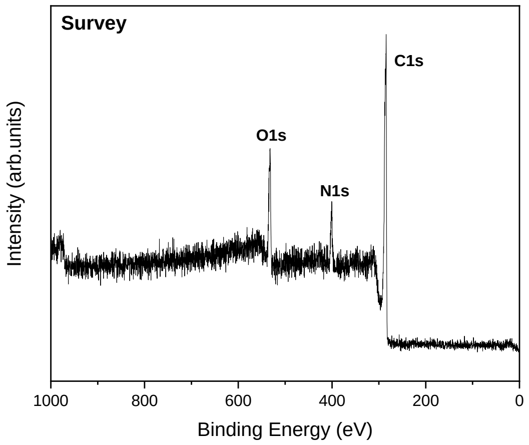

Figure S1. XPS survey spectrum of bio-Graphene.

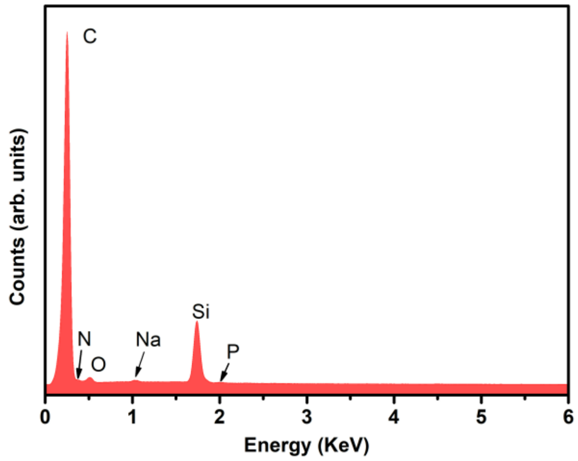

Figure S2. EDX spectrum of the tri-enzymatic system immobilized on bio-Graphene.

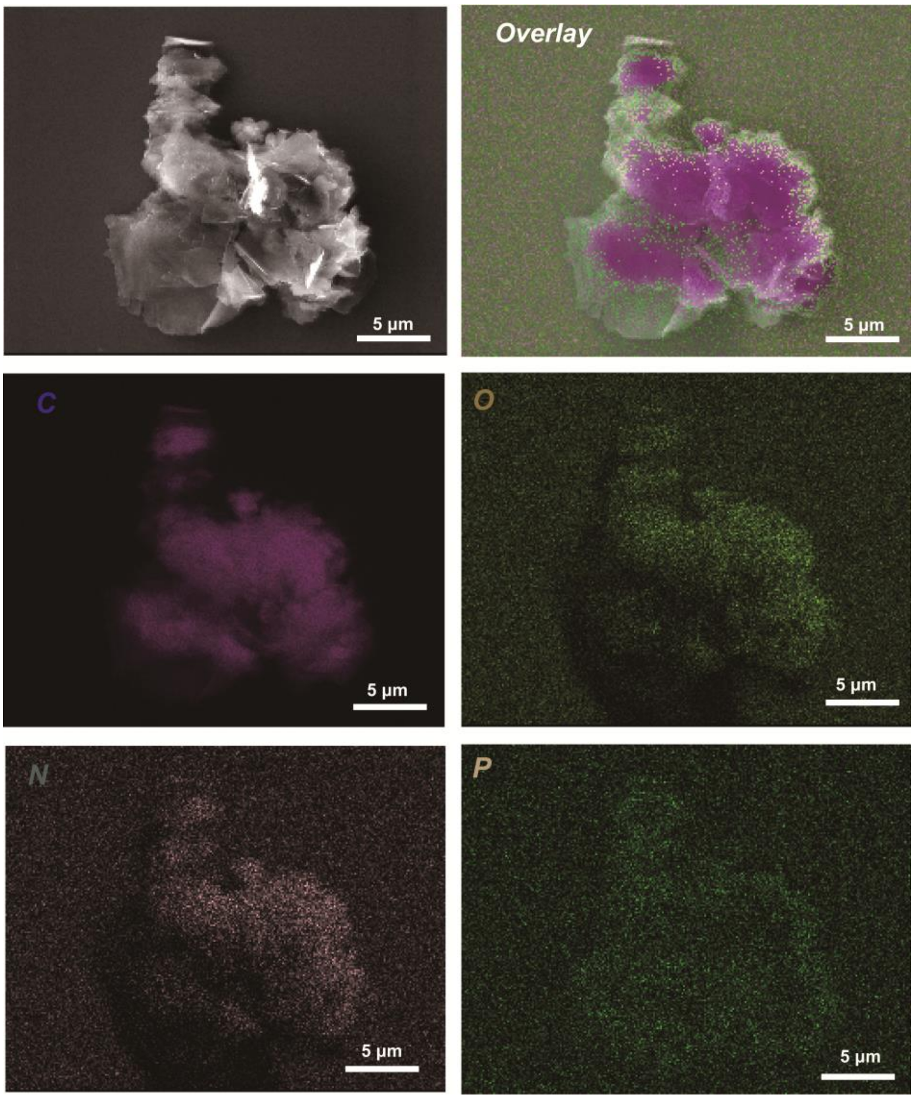

Figure S3. SEM image (top left panel), and EDX mapping of C (middle left panel), O (middle right panel), N (bottom left panel) and P (bottom right panel) as well as overlay (top right panel) of the maps of a flake of tri-enzymatic system immobilized on bio-Graphene.

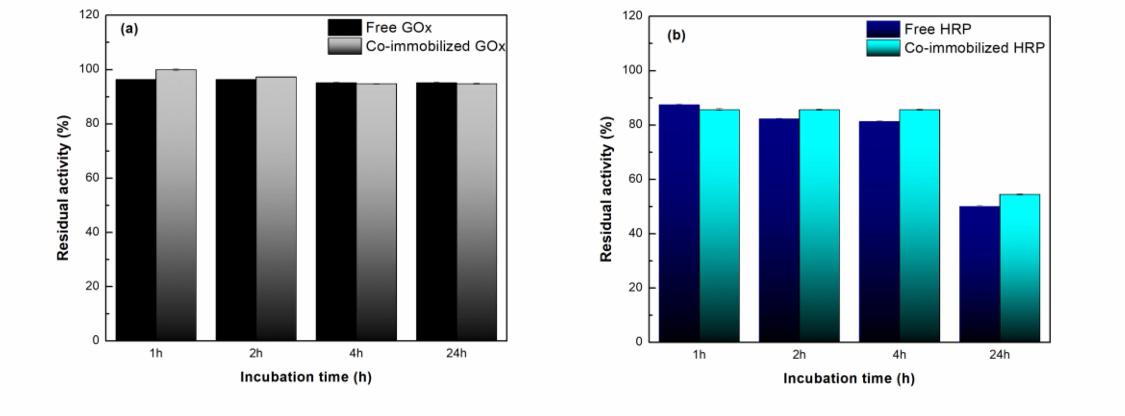

Figure S4. Stability of free and co-immobilized GOx and HRP on bio-Graphene: residual activity after incubation at 37 °C for different times.

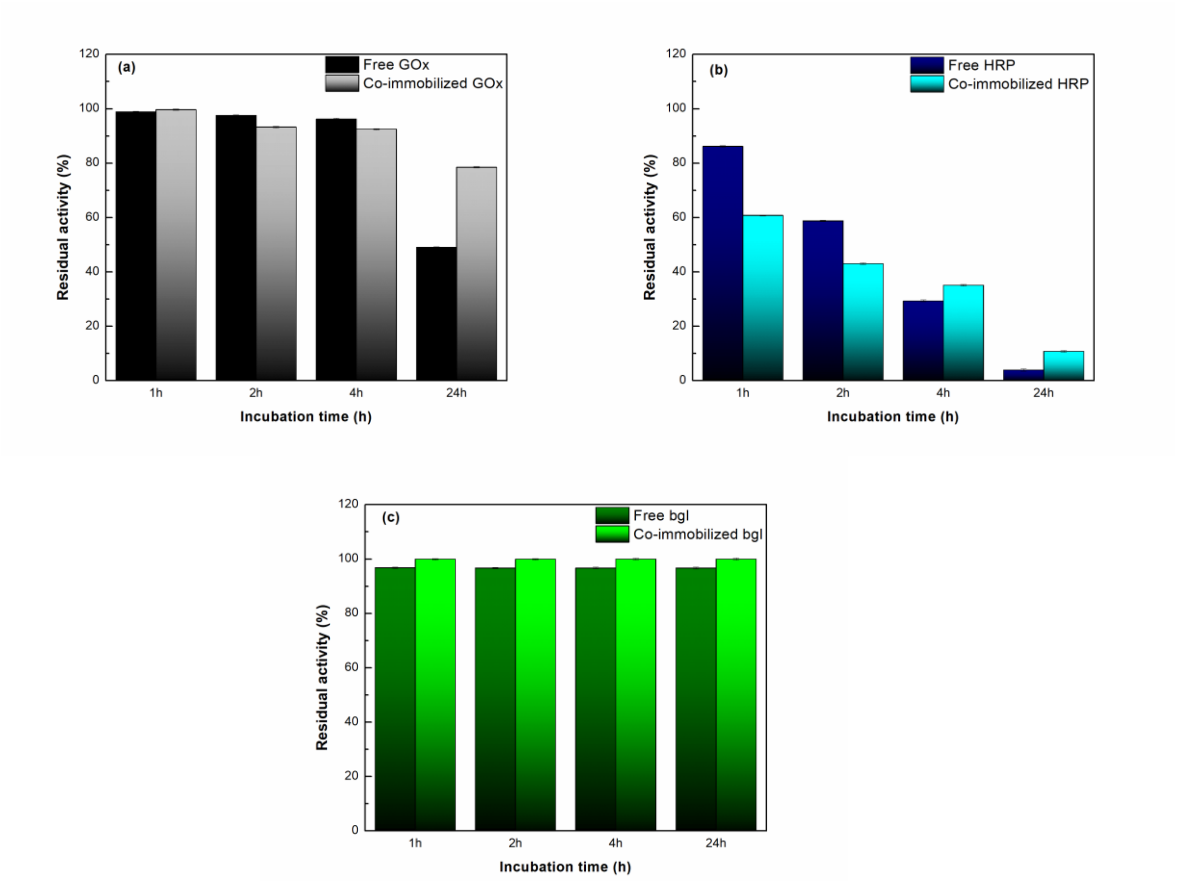

Figure S5. Stability of free and co-immobilized GOx, HRP, and bgl on bio-Graphene: residual activity after incubation at 50 °C for different times.

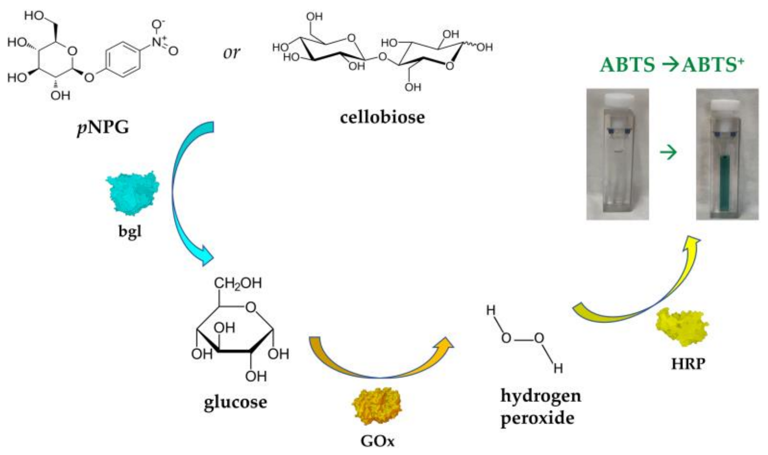

Figure S6. Schematic presentation of the cascade reaction of *p*NPG and cellobiose hydrolysis to $ABTS^+$, catalyzed by the tri-enzymatic nanobiocatalyst.

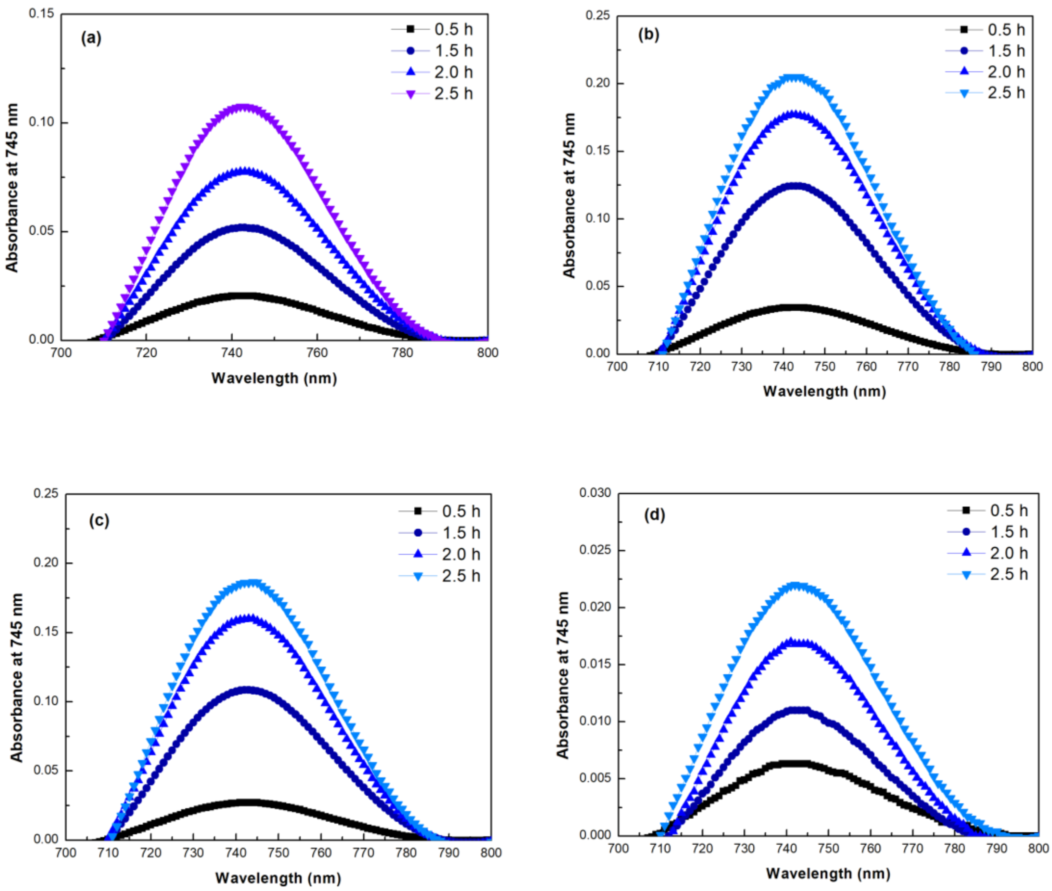

Figure S7. Time-dependent increase in absorption intensity at 745 nm for the cascade reaction of *p*NPG hydrolysis to $ABTS^{+}$, catalyzed by the tri-enzymatic nanobiocatalyst at a) 25 ^{o}C, b) 30 ^{o}C, c) 40 ^{o}C, and d) 50 ^{o}C.
